# Adult human cardiac stem cell supplementation effectively increases contractile function and maturation in human engineered cardiac tissues

**DOI:** 10.1186/s13287-019-1486-4

**Published:** 2019-12-04

**Authors:** Jack F. Murphy, Joshua Mayourian, Francesca Stillitano, Sadek Munawar, Kathleen M. Broughton, Esperanza Agullo-Pascual, Mark A. Sussman, Roger J. Hajjar, Kevin D. Costa, Irene C. Turnbull

**Affiliations:** 1 Cardiovascular Research Center, Icahn School of Medicine at Mount Sinai, One Gustave L. Levy Pl, Box 1030, New York, NY 10029 USA; 2 San Diego Heart Research Institute, San Diego State University, San Diego, USA; 3 Microscopy Core, Dean’s CoREs, Icahn School of Medicine at Mount Sinai, New York, USA; 4 Phospholamban Foundation, Amsterdam, The Netherlands

**Keywords:** Human cardiac stem cells, Human engineered cardiac tissue, Contractility, Myocardial function, Cardiomyocyte maturation

## Abstract

**Background:**

Delivery of stem cells to the failing heart is a promising therapeutic strategy. However, the improvement in cardiac function in animal studies has not fully translated to humans. To help bridge the gap between species, we investigated the effects of adult human cardiac stem cells (hCSCs) on contractile function of human engineered cardiac tissues (hECTs) as a species-specific model of the human myocardium.

**Methods:**

Human induced pluripotent stem cell-derived cardiomyoctes (hCMs) were mixed with Collagen/Matrigel to fabricate control hECTs, with an experimental group of hCSC-supplemented hECT fabricated using a 9:1 ratio of hCM to hCSC. Functional testing was performed starting on culture day 6, under spontaneous conditions and also during electrical pacing from 0.25 to 1.0 Hz, measurements repeated at days 8 and 10. hECTs were then frozen and processed for gene analysis using a Nanostring assay with a cardiac targeted custom panel.

**Results:**

The hCSC-supplemented hECTs displayed a twofold higher developed force vs. hCM-only controls by day 6, with approximately threefold higher developed stress and maximum rates of contraction and relaxation during pacing at 0.75 Hz. The spontaneous beat rate characteristics were similar between groups, and hCSC supplementation did not adversely impact beat rate variability. The increased contractility persisted through days 8 and 10, albeit with some decrease in the magnitude of the difference of the force by day 10, but with developed stress still significantly higher in hCSC-supplemented hECT; these findings were confirmed with multiple hCSC and hCM cell lines. The force-frequency relationship, while negative for both, control (− 0.687 Hz^− 1^; *p* = 0.013 vs. zero) and hCSC-supplemented (− 0.233 Hz^− 1^;*p* = 0.067 vs. zero) hECTs, showed a significant rectification in the regression slope in hCSC-supplemented hECT (*p* = 0.011 vs. control). Targeted gene exploration (59 genes) identified a total of 14 differentially expressed genes, with increases in the ratios of *MYH7/MHY6*, *MYL2/MYL7*, and *TNNI3/TNNI1* in hCSC-supplemented hECT versus controls.

**Conclusions:**

For the first time, hCSC supplementation was shown to significantly improve human cardiac tissue contractility in vitro, without evidence of proarrhythmic effects, and was associated with increased expression of markers of cardiac maturation. These findings provide new insights about adult cardiac stem cells as contributors to functional improvement of human myocardium.

## Background

Heart failure (HF) is a worldwide public health problem, with an estimated prevalence of 2% for the overall adult population [1]. To curtail this growing global health crisis [2, 3], development of therapies to prevent, mitigate progression, or reverse HF, is of the utmost importance.

One promising therapeutic strategy involves the delivery of stem cells to the failing heart, which has resulted in significant improvements of cardiac structure and function in multiple preclinical animal studies [4, 5]. Unfortunately, this success in animals has not fully translated to humans. In clinical trials, while most stem cell therapies have proven to be safe, the evidence of efficacy remains controversial, with contractile benefits that have often been modest in effect and transient in duration [6, 7]. Indeed, a recent meta-analysis based on individual patient data reported no effect of stem cell therapy on left ventricular function or clinical outcome [8], which may partly reflect inconsistencies in cell type, dosage, method of delivery, and clinical condition. Nonetheless, there remains strong interest, including active clinical trials, aiming to identify the ideal stem cell type and dose for optimizing the treatment of myocardial dysfunction [9, 10].

Challenges with the clinical translation of stem cell therapy for the heart indicate an incomplete understanding of the underlying biological mechanisms involved. For example, multiple animal studies from independent investigative teams have shown that cardiac stem cells (CSCs), identified by positive expression of the stem cell growth factor receptor CD117 (or c-Kit), show improvement in cardiac function when delivered after myocardial injury [11–13]. However, while the purported cardiomyogenic ability of these CSCs appears to be functionally insignificant, if not nonexistent [14, 15], evidence suggests that CSCs can exert other beneficial effects on the myocardium through paracrine mechanisms [16, 17]. Further investigation of the underlying mechanisms of action, which are understood to be complex and multifactorial [16], is required to fully exploit the cardiotherapeutic potential of adult CSCs.

Limited access to suitable experimental models of the human heart has exacerbated these gaps in mechanistic knowledge. However, human pluripotent stem cells now provide a nearly limitless supply of differentiated human cardiomyocytes [18, 19], and use of these cells to create 3-D human engineered cardiac tissues (hECTs) allows direct measurement of twitch force and related characteristics of cardiac muscle contractility [20]. Recognized challenges with the maturation status of stem cell-derived cardiomyocytes [21, 22] may limit their suitability for direct clinical applications like surgical implantation. However, the resulting engineered tissue constructs have been shown to reproduce key features of cardiac muscle physiology [20, 23], to predict human cardiac responses to drug interventions for in vitro toxicology screening [24, 25], and to recapitulate phenotypic characteristics of human heart diseases for developing new therapeutics [26–29]. In the context of studying stem cell therapies, our group has previously used hECTs to show that paracrine signaling, and not heterocellular coupling, is primarily responsible for the pro-contractile effects of human mesenchymal stem cells (hMSCs) on human cardiomyocytes [30], and to identify a specific exosomal microRNA that mediates hMSC-induced cardiac contractile enhancement [31]. For such studies, hECTs are well suited to isolate effects on contractility independent of concomitant processes such as immune suppression, neovascularization, and endogenous repair mechanisms that often complicate the interpretation of in vivo animals, with the added advantage of human species specificity studies.

In contrast to hMSCs, the specific effects of human CSCs (hCSCs) on the contractile performance of host human myocardium remain poorly understood, limiting efforts to optimize therapeutic efficacy. In this study, we used our well-established hECT platform to specifically examine the effects of primary adult hCSCs, isolated from diseased adult left ventricle apex biopsy, on human cardiac contractile force for the first time in vitro, as a step toward improving the safety and efficacy of stem cell-based cardiotherapies for patients.

## Methods

### Cell sources and human engineered cardiac tissue (hECT) fabrication

The human cells used in this study were obtained without involving intervention or interactions with the individuals, and the information was not individually identifiable; in agreement with Human Subject Regulations Decision Charts from the Office for Human Research Protections (OHRP) of the Department of Health and Human Services (HHS), this research is not research involving human subjects and 45 CFR part 46 does not apply. The hECT were fabricated using human induced pluripotent stem cells (hiPSCs) from a healthy cell line (SKiPS-31.3) [32], which were differentiated into cardiomyocytes following an established monolayer-based directed differentiation protocol by the addition of small molecules to induce temporal modulation of Wnt signaling [18], modified using CHIR99021 (10 μM concentration, Selleckchem) for days 0–1, and the addition of IWR-1 (5 μM concentration, Sigma) on days 3 and 4 [27]. The hiPSC-derived cardiomyocytes (hCMs) were collected, after 24–29 days of differentiation, by enzymatic digestion of the hCM monolayer (trypsin 0.025%), and counted after centrifugation; the hCM cell pellet was resuspended in a solution of type-I collagen and Matrigel (to yield 1 million cells per hECT), and then pipetted into custom multi-tissue bioreactors consisting of a removable Teflon-based plate with six wells that hold the cell-matrix solution, and a silicone (polydimethylsiloxane, PDMS) rack system with six pairs of flexible posts that form anchors during formation of up to six tissues and act as integrated force sensors that deflect during hECT beating, as we have recently described in detail [27].

For fabrication of the hECTs supplemented with human cardiac stem cells (hCSCs), we used hCSCs that were isolated, characterized, and expanded at the San Diego Heart Research Institute (San Diego State University, CA) as described in a detailed methods paper [33]. In brief, left ventricular assist device (LVAD) implantation explant cardiac tissue was minced, then digested in collagenase type II (Worthington-Biochem, Lakewood, NJ) for approximately 2 h at 37 °C. Cells were pipetted under the hood to further separate cells from extracellular matrix and spun down at 700 rpm for 2 min to pellet cardiomyocytes. Supernatant was collected and spun down at 1200 rpm for 5 min to pellet interstitial cells. Cardiac interstitial cells were filtered through a 100- and 40-μm filter then resuspended in hCSC media (F12 HAM’s (1×), 10% ES FBS, 1% Penicillin-Streptomycin-Glutamine (100×), 5 mU/mL human erythropoietin, 10 ng/mL human recombinant basic FGF, and 0.2 mM l-Glutathione), and plated overnight; the following day, cells were collected from the media and tryptased from the plate with single cells incubated with microbeads conjugated to tyrosine-protein kinase Kit (c-Kit; CD117 Human MicroBead, Miltenyibiotec, Auburn, CA) and magnetically sorted. A portion of the c-Kit^+^ cells, obtained from bead sorting, were cultured and expanded in hCSC media. Cells were expanded and frozen back in freezing media (45% hCSC media, 45% ES FBS, 10% DMSO). After passage 5, the cells were shipped frozen to the Cardiovascular Research Center (Icahn School of Medicine at Mount Sinai, NY), where they were thawed and cultured in hCSC media (described above)). To split hCSCs, cells were washed with 1× PBS two to three times and incubated for 5 min (37 °C, 5%CO_2_) in dissociation media composed of 1:1 mix of CellStripper Dissociation Reagent (Corning) and TrypLE Express Enzyme (Thermo Fisher); after confirmation by visualization under the microscope that the cells were lifted from the dish, an equal amount of defined media (described above) was added; and the cells, along with dissociation media and defined media, were transferred to a conical tube for centrifugation (300*g* × 5 min). Then the supernatant was discarded, and the cells were resuspended in defined media, counted, and plated. We used hCSCs at passages 7–12 for hECT fabrication. On the day of hECT fabrication, both hCM and hCSC were collected separately and counted, and then the cells were distributed in conical tubes containing either hCM only, or hCM + hCSC at a 9:1 ratio, so that both hCM-only control hECTs and the hCSC-supplemented hECTs contained the same total cell number. The hECT were incubated at 5% CO_2_ and 37 °C and maintained in RPMI/B27 + Insulin media with daily half-media exchanges. The hECTs were checked daily for compaction and spontaneous beating under bright field microscopy starting at 48 h after fabrication, when bioreactor base plates were removed.

For our first studies, we used CSCs derived from a 67-year-old male patient with advanced heart failure. Then, we performed further functional experiments fabricating hECT with hCMs from a second hiPSC healthy cell line (MSN02–4) and tested the first hCSC cell line and then a second hCSC cell line derived from a 59-year-old male patient, also with advanced heart failure. For the additional cell lines, the functional experiments were performed as described above and included higher pacing frequencies, up to 2 Hz, also with 0.25-Hz increments.

### Analysis of contractile function

Starting on day 6, the contractile function of each hECT was measured in a laminar flow hood in a custom setup that allows real-time tracking of PDMS post deflection versus time using custom LabVIEW software to acquire a 30-s data sampling. The data is then analyzed with a custom MATLAB script to calculate twitch parameters including developed force (DF; calculated from the post deflection with each twitch), maximum contraction rate (+dF/dt), maximum relaxation rate (−dF/dt), developed stress (DS; DF divided by hECT cross-sectional area), and passive force (resting force between each twitch, assessed from the post deflection referenced to the initial position at the time of tissue fabrication), as previously described [27]. Recordings were first performed without electrical stimulation to obtain data on spontaneous beating frequency and spontaneous beat rate variability, measured as the coefficient of variation (COV) of individual twitches over the 30-s acquisition time window. However, because the force exerted by hECTs is dependent on beating frequency, all comparisons related to twitch force parameters were performed under controlled pacing conditions. The hECTs were electrically paced by field stimulation (12-V biphasic pulse with 5-ms duration) at increasing frequencies from 0.25 to 1.0 Hz with 0.25-Hz increments, using a programmable Grass S88X stimulator (Astro-Med, West Warwick, RI). At the end of the testing session, hECTs were rinsed once with 1× PBS, then the bioreactor was replenished with fresh culture media (RPMI 1640, B-27 Supplement (50×), 1% penicillin-streptomycin) and returned to the incubator (5%CO_2_/37 °C), for further testing at additional time points (day 8 and day 10).

### Immunofluorescence

For immunofluorescence, immediately following functional measurements on day 10, the hECTs were rinsed in 1× phosphate-buffered saline (PBS) and fixed with 4% paraformaldehyde for at least 24 h at 4 °C. To perform whole mount staining, fixed hECTs were incubated in PBS/0.5% Triton X-100 for 90 min, with fresh triton solution replaced every 30 min, incubated in 10% goat serum for 2 h with fresh goat serum solution replaced after the first hour, incubated in primary antibodies diluted in antibody diluent (DAKO, Agilent) at 4 °C for 4 days, while maintained on a tube rotator. Then the tissues were washed for 3 h, with fresh buffer solution replaced every hour, using a buffer solution that was composed of 50% Triton X-100 0.5% in PBS, and 50% goat serum 5%. The hECTs were incubated in secondary antibody diluted in DAKO solution at 4 °C for 4 days, while maintained on a tube rotator and protected from light. The hECTs were washed in PBS three times for 1 h each. Primary antibodies were monoclonal anti-α-actinin (sarcomeric) (1:200, A7811, Sigma-Aldrich) and cardiac Troponin T (1:200, clone 13–11 Invitrogen MA512960); secondary antibodies were AF594 goat anti-mouse (1:200, A11032, Life Technologies) and goat anti rabbit AF 488 (1:200, A11034, Life Technologies); nuclei stained with either DAPI or anti-histone H2B (1:200, SAB4502228, Sigma-Aldrich). For two-photon microscopy analysis, the hECTs were then placed on a glass slide in PBS, covered with a 1.5-mm glass slide. Two-photon microscopy was performed using an Olympus FV1000 MPE multi-photon laser scanning microscope. For confocal microscopy, the hECTs were placed on an imaging dish (ibidi 80136) in PBS, covered with a 1.5-mm glass slide. Confocal microscopy was performed using a Zeiss LSM 880 microscope. Image analysis was performed using Fiji software [34].

### hCSC labeling

In order to track the hCSCs within the hECTs, we labeled the hCSCs prior to hECT fabrication using a Qtracker 655 labeling kit (Q25029 Thermo Fisher Scientific) for labeling suspension cells. On the day of hECT fabrication, we collected the hCSCs with the protocol described above using dissociation media composed of 1:1 mix of CellStripper Dissociation Reagent (Corning) and TrypLE Express Enzyme (Thermo Fisher); after counting the cells, we set aside an aliquot of 2 million cells in 200 μl of culture media and proceeded with mixing components A and B of the kit, following the manufacturer’s instructions. After adding the cells to the mix, we incubated the cells for 60 min maintaining them in a 1.5-ml microcentrifuge tube in rocking motion at 37 °C. After incubation, the cells were washed twice. We took an aliquot of the cells to plate and confirm the Qtracker labeling with fluorescence microscopy and took another aliquot of labeled hCSCs with which we proceeded to use them for the cell/hydrogel mix at the prescribed ratio of 9hCM:1hCSC for the fabrication of hCSC-supplemented hECTs. These tissues were then used to track the hCSCs within the hECTs. After day 10, we fixed the tissues and performed whole tissue stain with DAPI, we performed confocal microscopy using a Zeiss LSM 880 microscope, and we obtained images for semiquantitative analysis of distribution and abundance of the hCSCs within the hECTs; using Fiji software [34], the hCSCs/total cell count (Qtracker/DAPI) percentage was obtained based on counting the number of particles.

### Live/dead staining

At the completion of the functional studies, the hECTs were washed with PBS and then placed in microcentrifuge tubes, where they were stained using a live/dead cell imaging kit (R37601 Invitrogen) following the manufacturer’s instructions. Images were obtained after 15 min-incubation on a confocal microscope (Zeiss LSM 880), and analysis was performed using a thresholded pixel/area measurement in Fiji software [34].

### RNA isolation

For genetic analysis, immediately following functional measurements on day 10, the hECTs were rinsed in 1× PBS, frozen, and stored at − 80 °C for future RNA extraction. For RNA extraction, hECTs were placed in lysing matrix tubes (MPbio 6913-500) with 600 μl of TRI Reagent (Zymo) and homogenized using a FastPrep-24 homogenizer (MPbio), for 30 s at a speed of 6.0 m/s; for two runs, tubes were incubated on ice for 5 min between each run. The samples were further processed using a Direct-zol RNA with on column DNase I treatment, following the manufacturer’s instructions. RNA concentration was measured using a Nanodrop 2000 spectrophotometer.

### Genetic analysis

mRNA levels were quantified using NanoString Technology. An nCounter custom codeset was designed for the identification of genes of interest related to cardiomyocyte contractility, maturation and apoptosis totaling 59 genes, plus 5 housekeeping genes (*B2M*, *EEF1A1*, *GAPDH*, *RNPS1*, and *SRP14*), selected based on published data for the selection of reference genes in human myocardium [35] and GENEVESTIGATOR [36] software. RNA inputs of 100 ng were used for hybridization and placed on a cartridge for the NanoString reader. The output files (RCC files) were loaded into nSolver Analysis software; after running the data for quality control and background normalization, genes of interest were normalized to the housekeeping genes. The output counts from the nSolver Analysis were normalized to cardiac-specific *TNNT2* and then reported as the fold change of hCSC-supplemented hECTs relative to hCM-only hECT control.

Partial least squares regression (PLSR) was performed with the nonlinear iterative partial least squares algorithm, as described elsewhere [31, 37] using Unscrambler® X (CAMO Software). PLSR was performed on the expression of select genes studied via Nanostring for both hCM-only hECT controls and hCSC-supplemented hECTs, matched to hECT responses of DF, DS, +dF/dt, −dF/dt, and several time characteristics during a contraction. Explained variance for input gene expression data is shown in the figures. Predictability of the trained model corresponds to the predicted vs. reference coefficient of determination.

### Statistical analysis

Descriptive statistics are reported as mean and standard deviation, with values reported as fold changes relative to hCM-only controls unless otherwise specified. Student’s *t* test was used for comparisons between the two groups of hECTs. Linear regression was used to test significance of the slope in the force-frequency analysis. Statistical analysis was performed using GraphPad Prism software. Statistical significance was accepted at the *p* < 0.05 level. Structural alignment was evaluated using MatFiber3 software and circular statistics to determine the mean angle and circular standard deviation (CSD) which ranges from 0° for perfectly aligned to 40.5° for random orientation [38]. Principal component analysis (PCA) and partial least squares regression (PLSR) were performed using the Camo Unscrambler suite from CamoAnalytics software.

## Results

### Structural and functional characteristics of hECT

**Fig. 1:**
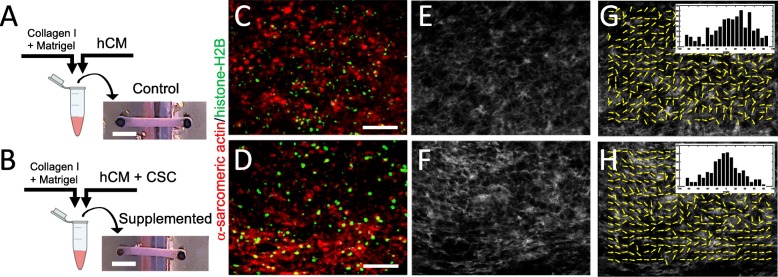
Fabrication of human engineered cardiac tissues (hECTs). **a** Diagram of fabrication of hECTs using a mix of hydrogel matrix (Collagen type-I plus Matrigel) and human induced pluripotent stem cell-derived cardiomyocytes (hCM). The solution is pipetted into a custom bioreactor with flexible end-posts, resulting in a self-assembled muscle strip-like shape within 72 h. **b** An identical approach was used to fabricate hCSC-supplemented hECTs, with the exception of adding cardiac stem cells (hCSCs) at a ratio of 90% hCM: 10% hCSCs. Scale bar of 2000 μm applies to **a** and **b**. **c**, **d** Two-photon images of control hECT (**c**) and supplemented hECT (**d**) stained for α-sarcomeric actin (red) and histone-H2B (green). Scale bar of 100 μm applies to panels **c**–**h**. **e**, **f** Second harmonic generation imaging of the corresponding two-photon image displaying collagen fibers, and **g**, **h** MatFiber3 analysis of collagen fiber orientation in images from **e** and **f**, with histograms of fiber alignment distribution (inset), showing mean angle ± CSD of 22.5° ± 33.5°, and − 2.95° ± 29.8°, respectively. Images were obtained with the hECTs oriented horizontally in the field of view

**Fig. 2:**
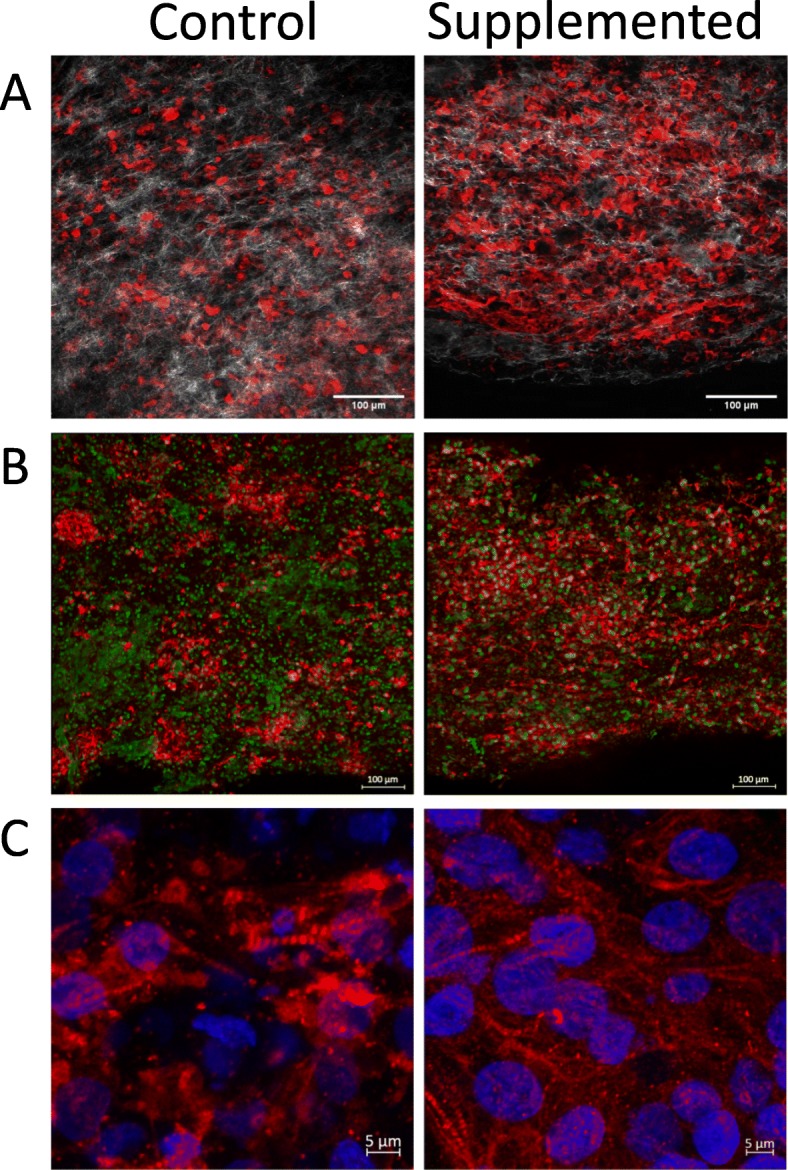
Organization and microstructure of hECTs. Representative microscopy images of hECTs obtained along the horizontal axis of the hECT. **a** Single slice image obtained on a two-photon microscope, showing alpha-actinin (red) and collagen fibers (white), scale bar = 100 μm. **b** Confocal max projection showing Cardiac Troponin T (red), and nuclei stained with anti-histone H2B (green), scale bar = 100 μm. **c** Confocal max projection showing myofibril organization, with cardiac Troponin T (red), and nuclei stained with DAPI (blue,) scale bar = 5 μm

The hECTs used in this study were fabricated from four different batches of differentiated hCMs, yielding 5–12 hECTs per batch (including both hCM-only controls and hCSC-supplemented hECTs). The hECTs from both groups compacted into a muscle strip-like shape within 72 h after pipetting the cell/hydrogel mix into the custom bioreactor [27] (Fig. 1a, b). The hECTs first displayed spontaneous beating by 72 h after fabrication (detected by visualization under light microscope with × 4 objective), and post deflection (detected by visualization in low magnification microscope × 2) day 6 after fabrication (as detailed below). Whole tissue mount staining for α-actinin (sarcomeric) antibody (as a cardiac marker) and histone-H2B (for identification of nuclei), imaged using two-photon fluorescence microscopy, showed distribution of cells positive for α-sarcomeric actin throughout the width and thickness of the tissue (Fig. 1c, d). Second harmonic generation imaging showed collagen fibers with a predominant alignment along the axial orientation of the hECT (Fig. 1e, f), with a narrower distribution in hCSC-supplemented hECTs, as quantified by measuring structural orientation in 7 × 7-pixel image subregions (Fig. 1g, h) with analysis by circular statistics using a published MatLAB script called MatFiber3 [38]. While there was no quantifiable evidence of differences in the overall cell distribution between hCM-only controls and hCSC-supplemented hECTs, the latter showed better organization, a more homogenous distribution of cells positive for cardiac markers (α-actinin and cardiac troponin T), with microstructural features showing myofibrils with tighter arrangement within the hECT (Fig. 2).

**Fig. 3:**
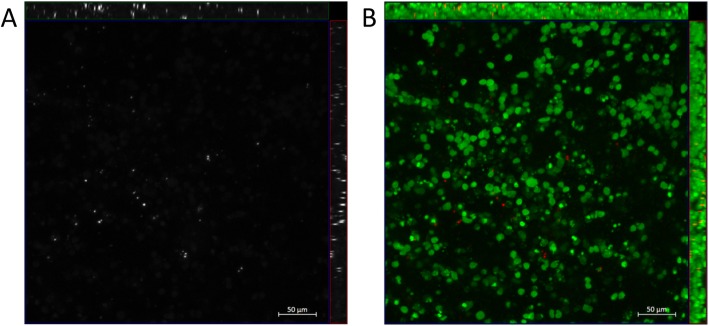
Distribution of hCSC within the hECTs. Representative image of hCSC-supplemented hECT fabricated with hCSCs that were labeled with Qtracker-655 prior to tissue fabrication. Confocal max projection with orthogonal projections in **a** grayscale image, Cy5 channel only, showing Qtracker-655-labeled cells (white), and **b** merged image showing Qtracker-655-labeled cells (pseudocolored red) and nuclei stained with DAPI (pseudocolored green)

To investigate the distribution of hCSCs within the hECTs, we fabricated a subset of hECTs where on the day of hECT fabrication, first we labeled the hCSC with Qtracker-655, and then we used them for the cell/hydrogel mix at the prescribed ration of 9hCM:1hCSC for the fabrication of hCSC-supplemented hECTs. Confocal microscopy revealed that the hCSCs were distributed throughout the hECTs, with no particular regional localization (Fig. 3) and with no evidence of proliferation, retaining a relatively constant abundance, corroborated through semiquantitative analysis measuring hCSCs (Qtracker positive cells) relative to total nuclei count (DAPI) (9.7 ± 2.5%). Live dead stain did not reveal differences in cell viability in control and hCSC-supplemented hECTs, with a dead/live ratio of 48 ± 7% and 32 ± 18% respectively (*p* = 0.34). Co-staining with cardiac troponin T did not show evidence of colocalization of troponin T expression with Q-tracker655 (Additional file 1: Figure S1), indicating the lack of evidence of differentiation of hCSCs into cardiomyocytes.

### Cardiac stem cells increase hECT contractile function

**Fig. 4:**
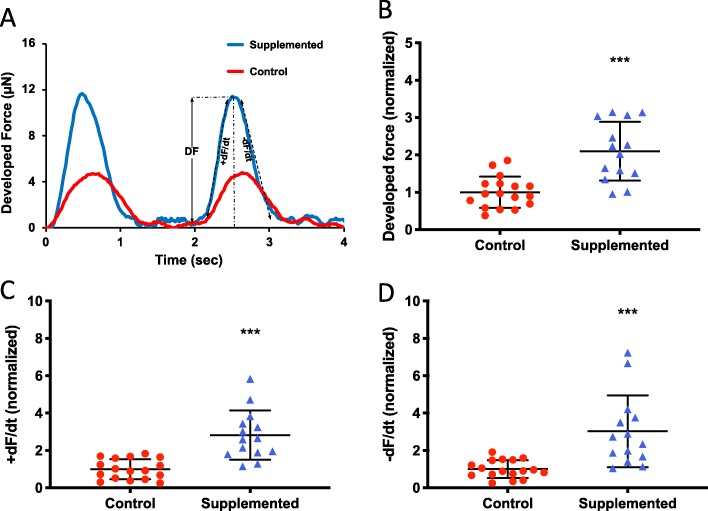
Twitch parameters under electrical stimulation. **a** Example twitch tracings for hCM-only control (red circle) and hCSC-supplemented hECTs (blue triangle) at 0.5-Hz electrical stimulation, illustrating the metrics of developed force (DF), rate of contraction (+dF/dt), and rate of relaxation (−dF/dt). **b** Developed force, **c** rate of contraction, and **d** rate of relaxation, were significantly higher in hCSC-supplemented (*n* = 17) vs. control (*n* = 14) hECTs. Measurements performed at 0.75-Hz pacing, and values normalized to mean control hECTs. Dot plots show individual data with bars representing mean ± SD; *** *p* < 0.001

**Fig. 5:**
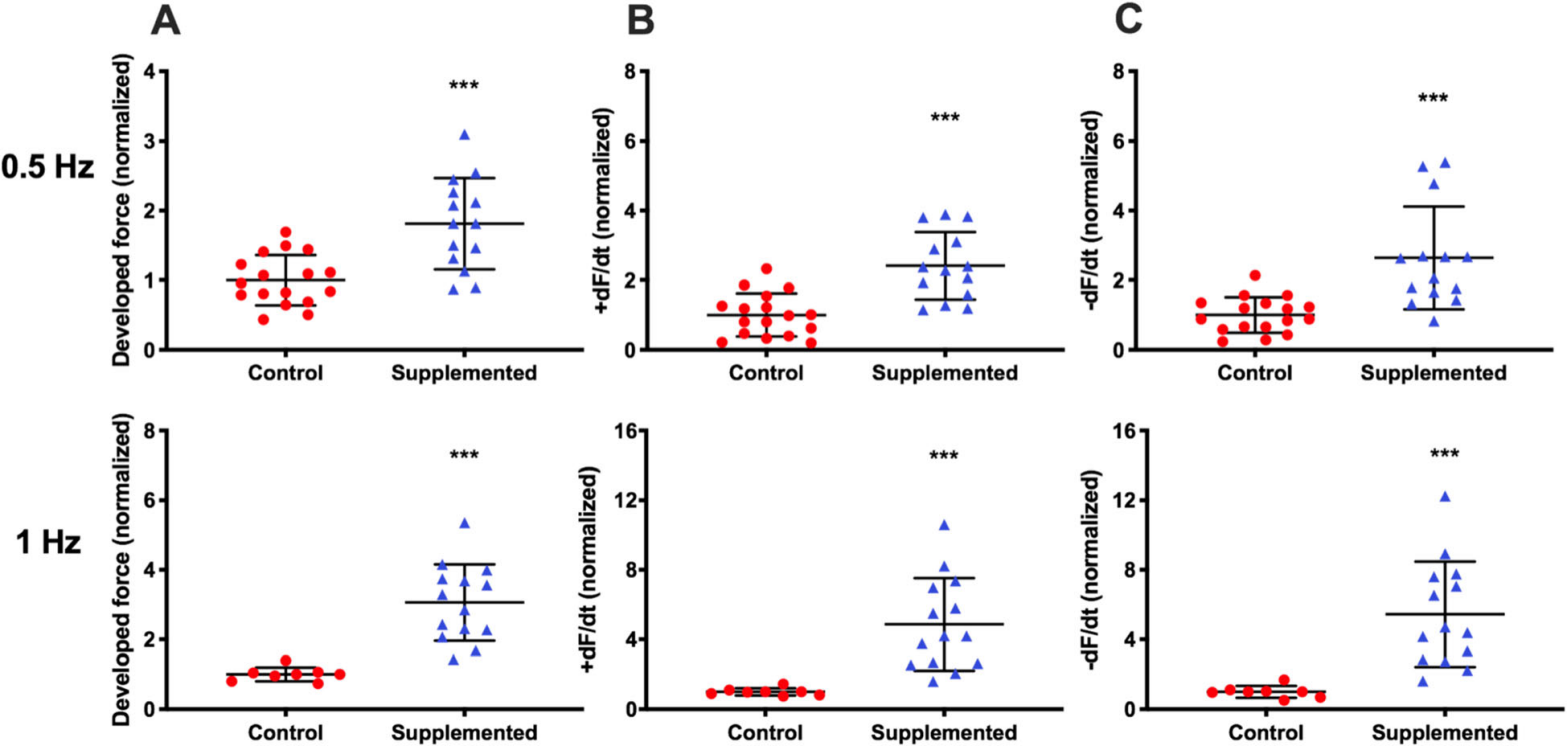
Contractile function parameters at 0.5-Hz and 1.0-Hz pacing on day 6**. a** Developed force, **b** rate of contraction (+dF/dt), **c** rate of relaxation (−dF/dt). For hCM-only controls (red circle), *n* = 17 at 0.5 Hz (left) and *n* = 8 at 1.0 Hz (right); for hCSC-supplemented hECTs (blue triangle), *n* = 14 at both pacing frequencies. Measurement values normalized to mean control hECTs. Dot plots show individual data with bars representing mean ± SD; ***p < 0.001

To evaluate the cardiac effects of hCSC supplementation, we measured the contractile function of the hCM-only controls and hCSC-supplemented hECTs (Fig. 4a). At day 6 of hECT fabrication, DF was approximately twofold higher in hCSC-supplemented hECTs (2.1 ± 0.79, *n* = 14) vs. hCM-only controls (1.0 ± 0.42; *p* < 0.0001, *n* = 17) during pacing at 0.75 Hz (Fig. 4b). Concomitantly, +dF/dt (Fig. 4c) and −dF/dt (Fig. 4d) were approximately threefold higher in the hCSC-supplemented hECTs (2.8 ± 1.32, *p* < 0.0001; and 3.0 ± 1.92, *p* = 0.0002, respectively). The hCSC-supplemented hECTs also displayed significantly higher DF, +dF/dt and −dF/dt than hCM-only controls at 0.5-Hz and 1.0-Hz pacing (Fig. 5).

**Fig. 6:**
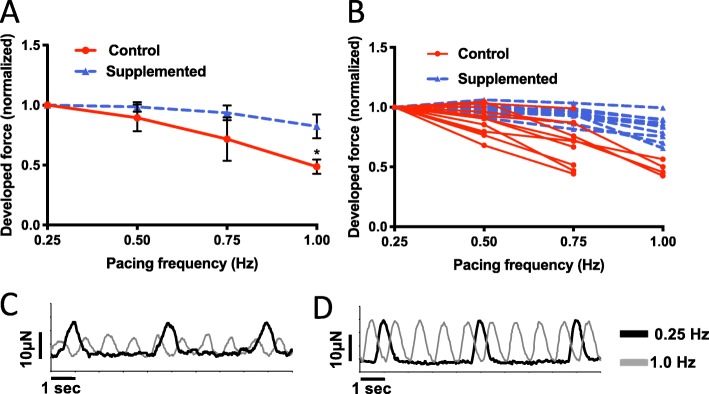
Force-frequency relationship. **a** Force-frequency response normalized to 0.25-Hz baseline for individual hECTs, with mean ± SD for control (red circle, *n* = 13) and hCSC-supplemented (blue triangle, *n* = 10) hECT groups. The regression slope for control hECTs was different from zero (*p* = 0.013) whereas the hCSC-supplemented slope was not (*p* = 0.067). **b** Force-frequency response of individual hECTs from hCM-only control (solid lines) and hCSC-supplemented (dashed lines) groups. hECTs that lost 1:1 capture below 1.0 Hz are indicated by abbreviated curves. **c, d** Example twitch tracings for hCM-only control (**c**) and hCSC-supplemented (**d**) hECTs at 0.25-Hz (black line) and 1.0-Hz pacing (gray line)

The direction and steepness of the force-frequency relationship is another measure of contractile function that also reflects the cardiac maturation status. For this analysis, we only included hECTs that captured at 0.25 Hz (*n* = 13 hCM-only and *n* = 10 hCSC-supplemented hECTs), and then recorded force tracings at increasing pacing frequencies of 0.5, 0.75, and 1.0 Hz or until 1:1 capture was lost; for slope comparison, DF was normalized to the value at 0.25 Hz for each hECT. The hCM-only control hECTs displayed a decreasing force-frequency relationship, with a significant regression slope of − 0.687 Hz^− 1^ (*p* = 0.013), reflecting the relative immaturity of the constituent stem cell-derived hCMs. In comparison, supplementation with hCSCs induced a rectification of the force-frequency curve (Fig. 6a) such that the force-frequency regression slope for hCSC-supplemented hECTs (− 0.233 Hz^− 1^) was not significantly different than zero (*p* = 0.067) and was significantly different from the control slope (*p* = 0.011). As noted above, 100% (*n* = 10 out of 10) of hCSC-supplemented hECTs exhibited 1:1 capture at all prescribed pacing frequencies, whereas only 31% (4/13) of the hCM-only control hECTs captured over the full frequency range (Fig. 6b). Example force tracings for control (Fig. 6c) and hCSC-supplemented (Fig. 6d) tissues paced at 0.25 Hz and 1.0 Hz further illustrate the difference in frequency dependence between these two groups of hECTs.

**Fig. 7:**
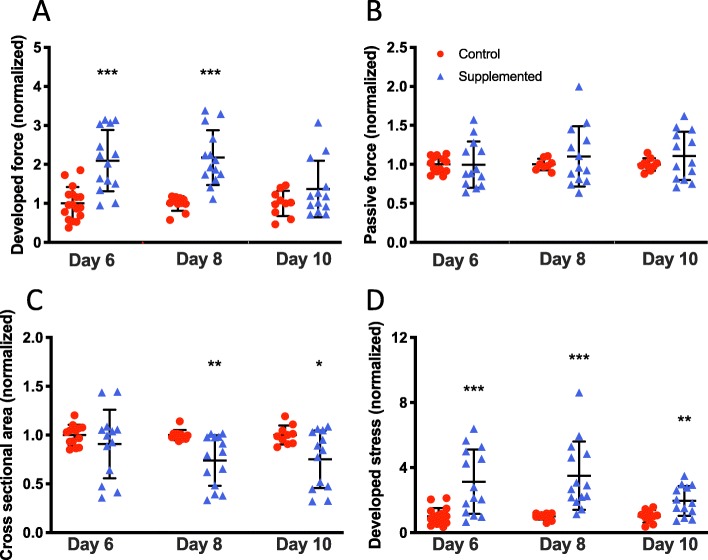
Time course of hECT twitch force. **a** Developed force at 0.75-Hz pacing at 6, 8, and 10 days after tissue fabrication for hCM-only control hECT (red circle) and hCSC-supplemented hECT (blue triangle). **b** Corresponding passive (diastolic) force from the same twitches analyzed for panel **a**. **c** Cross-sectional area of the hECT calculated from tissue diameter measured by digital image analysis immediately before twitch testing. **d** Developed stress (force/area) calculated from data in panels **a** and **c**. Dot plots show individual data with bars representing mean ± SD; all values normalized to mean hCM-only control at corresponding time point; *p < 0.05, **p < 0.01, ***p < 0.001

Functional characterization of hECTs was followed up on days 8 and 10, with values normalized to control for each day of analysis. The hCSC-supplemented hECTs retained a significantly higher DF than control on day 8 (2.18 ± 0.70 vs. 1.0 ± 0.19; *p* < 0.0001) at 0.75 Hz pacing, and this trend persisted to day 10 (1.37 ± 0.72 vs. 1.0 ± 0.32; *p* = 0.15) (Fig. 7a). We found that the average passive force for the hCSC-supplemented tissues remained within 10% of the value for hCM-only control hECTs at all three time points, with no statistically significant differences between groups (0.32 < *p* < 0.98), suggesting the reduced difference in developed force with time was not attributable to changes in passive force between hCSC-supplemented vs. control (Fig. 7b).

Compaction of the hECTs was evaluated by analyzing the cross-sectional area (CSA) calculated from measurements of tissue diameter. On day 6, the average CSA was slightly but not significantly smaller in hCSC-supplemented hECTs vs. hCM-only controls (0.91 ± 0.35 vs. 1.0 ± 0.11; *p* = 0.37), but compaction continued and reached statistical significance on days 8 and 10, with the hCSC-supplemented hECTs having a roughly 25% smaller CSA vs. hCM-only controls (day 8: 0.74 ± 0.26 vs. 1.0 ± 0.05, *p* = 0.003; day 10: 0.74 ± 0.26 vs. 1.0 ± 0.05, *p* = 0.019) (Fig. 7c). This higher degree of compaction combined with elevated DF translated into higher developed stress in hCSC-supplemented hECTs that was significantly greater than hCM-only controls at all time points (day 6: 3.13 ± 1.99 vs. 1.0 ± 0.51, *p* = 0.0002; day 8: 3.51 ± 2.10 vs. 1.0 ± 0.19, *p* = 0.0007; day 10: 1.96 ± 0.92 vs. 1.0 ± 0.38, *p* = 0.0058) (Fig. 7d).

### Cardiac stem cells preserve intrinsic beat rate characteristics of hECTs

**Fig. 8:**
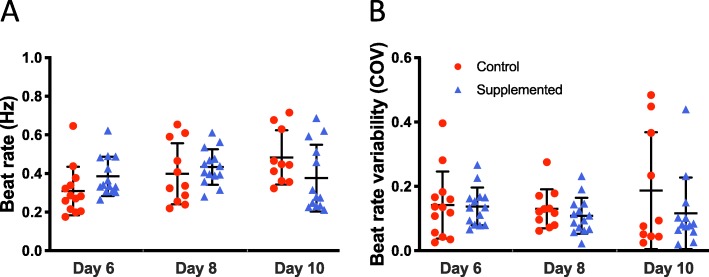
Intrinsic beat rate characteristics. **a** Spontaneous beat rate of hECTs on days 6, 8, and 10 after tissue fabrication. **b** Beat rate variability (measured as coefficient of variation, COV), at the same three time points. Dot plots show individual data with bars representing mean ± SD; *n* = 13, 11, 10 for hCM-only control hECT (red circle) and *n* = 14, 14, 13 for hCSC-supplemented hECT (blue triangle) on days 6, 8, and 10, respectively

The intrinsic beat rate characteristics of the hECTs were analyzed by comparing the spontaneous beat rate and its variability (COV) on days 6, 8, and 10 after tissue fabrication. The hECTs included in this analysis were those that demonstrated post deflection, detectable with low magnification microscope system, for video tracking without electrical stimulation, which on day 6 was true for all hCSC-supplemented hECTs (*n* = 14 of 14) but only 76% (*n* = 13 of 17) of hCM-only controls. The intrinsic (spontaneous) beat rate of hCSC-supplemented hECTs was similar to that of hCM-only controls at all time points (day 6: 0.39 ± 0.10 Hz vs. 0.31 ± 0.13 Hz; day 8: 0.43 ± 0.10 Hz vs. 0.40 ± 0.16 Hz; day 10: 0.38 ± 0.17 Hz vs. 0.48 ± 0.14 Hz), with no significant differences between groups (0.09 < *p* < 0.49) (Fig. 8a). The beat rate variability was also similar between the two groups of hECTs, with a trend toward slightly lower variability for the hCSC-supplemented vs. hCM-only controls at matched time points (day 6: 0.14 ± 0.06 vs. 0.14 ± 0.10; day 8: 0.11 ± 0.06 vs. 0.13 ± 0.06; day 10: 0.12 ± 0.11 vs. 0.19 ± 0.18) (Fig. 8b), though the trends were not statistically significant (0.36 < *p* < 0.89).

### Effects of cardiac stem cells on hECT gene expression profile

**Fig. 9:**
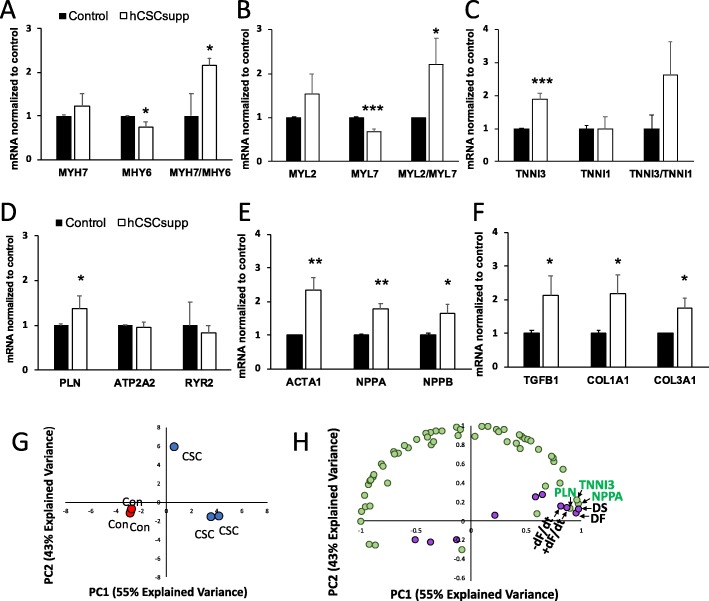
Gene expression analysis. Results from Nanostring Gene Assay, presented as mRNA transcript level in hCSC-supplemented hECTs (white bars) normalized to hCM-only controls (black bars) for genes associated with cardiac development/maturation (**a**, **b**, **c**), calcium handling (**d**), cardiomyogenic genes activated in response to stress (**e**), and extracellular matrix regulation (**f**). Bars represent mean ± SD; *n* = 3 hECT per group, *p < 0.05, **p < 0.01, ***p < 0.001. **g** Principal component analysis; red marks indicate hCM-only control (CON), blue marks indicate hCSC-supplemented hECTs (hCSCs) (*n* = 3 hECT per group). **h** Correlation loading plot from partial least squares regression suggests several significantly upregulated genes or downregulated genes that covary with hECT developed force, developed stress, +dF/dt, and −dF/dt (R 0.92); genes indicated by green markers, twitch parameters indicated by purple markers. DF = developed force, DS = developed stress, +dF/dt = maximum rate of contraction, and −dF/dt = maximum rate of relaxation

Gene expression analysis was carried out using a Nanostring gene expression custom assay on RNA isolated from samples frozen after study termination on day 10 to investigate the molecular effects resulting from supplementing the hECTs with hCSCs. Differentially expressed genes (DEGs) in hCSC-supplemented hECTs (vs. hCM-only controls) were quantified by the fold-change relative to controls and organized by their association with myocardial development and function. Of the 59-gene assay, hCSC supplementation significantly upregulated 8 genes and significantly downregulated 6 genes (see Table 1). For further analysis, the DEGs were grouped by biological function. Those associated with cardiomyocyte maturation were either upregulated or downregulated in hCSC-supplemented hECTs in trends that support increased maturation when compared to hCM-only controls; the average level of *MYH7* was 1.24-fold higher while *MYH6* was 0.74-fold lower, with a *MYH7/MHY6* ratio of 2.14 (Fig. 9a). In addition, *MYL2* was 1.53-fold higher, while *MYL7* was 0.69-fold lower, with an average *MYL2/MYL7* ratio of 2.21 (Fig. 9b). Lastly, *TNNI3* was 1.9-fold higher while *TNNI1* was relatively unchanged at 0.98×, yielding an average *TNNI3/TNNI1* ratio of 2.62 (Fig. 9c). For genes associated with calcium handling, hCSC supplementation had minimal effects on ATP2A2 (0.95×) and RYR2 (0.82×), while having a greater and statistically significant upregulatory effect on PLN (1.37×) (Fig. 9d). Cardiomyogenic genes, activated in response to stress, that were significantly upregulated by hCSC supplementation were *ACTA1* (2.35×), *NPPA* (1.78×), and *NPPB* (1.64×) (Fig. 9e). Extracellular matrix-related DEGs significantly upregulated in hCSC-supplemented hECTs were *TFGB1* (2.14×), *COL1A1* (2.18×), and *COL3A1* (1.74×) (Fig. 9f). Other significant DEGs, all downregulated relative to hCM-only control hECTs, were *AGTR1* (0.34×, *p* < 0.05), *HCN1* (0.40×, *p* < 0.01), *CDK1* (0.39×, *p* < 0.01), and *CASP3* (0.79×, *p* < 0.05), as listed in Table 1.

**Table 1:** List of genes included in NanoString Codeset

Gene symbol	Gene name	Fold change*	*p* value
*ACTA1*	Actin, Alpha 1, Skeletal Muscle	2.35	0.0030
*ACTA2*	Actin, Alpha 2, Smooth Muscle, Aorta	1.17	0.3664
*ACTN2*	Actinin Alpha 2	1.00	0.9877
*ADRB1*	Adrenoceptor Beta 1	1.39	0.2645
*ADRB2*	Adrenoceptor Beta 2	1.35	0.2586
*ADRB3*	Adrenoceptor Beta 3	0.84	0.7008
*AGTR1*	Angiotensin II Receptor Type 1	0.34	0.0214
*ATP2A2*	ATPase Sarcoplasmic/Endoplasmic Reticulum Ca2+ Transporting 2	0.95	0.6659
*BAX*	BCL2 Associated X, Apoptosis Regulator	1.11	0.6011
*BCL2*	BCL2, Apoptosis Regulator	0.57	0.1837
*BCL2L1*	Apoptosis Regulator Bcl-X	1.08	0.7545
*BCL2L2*	Apoptosis Regulator Bcl-W	0.88	0.5770
*CACNA1C*	Voltage-Gated L-Type Calcium Channel Cav1.2 Alpha 1 Subunit	1.06	0.8099
*CASP3*	Caspase 3	0.79	0.0175
*CASP8*	Caspase 8	0.70	0.4188
*CASP9*	Caspase 9	1.05	0.8472
*CASQ2*	Calsequestrin 2	0.49	0.0902
*CD34*	CD34 Molecule	1.15	0.1137
*CD44*	CD44 Molecule (Indian Blood Group)	2.28	0.2705
*CDK1*	Cyclin Dependent Kinase 1	0.39	0.0043
*CNTN2*	Contactin 2	0.53	0.2429
*COL1A1*	Collagen Type I Alpha 1 Chain	2.18	0.0254
*COL3A1*	Collagen Type III Alpha 1 Chain	1.74	0.0151
*CTGF*	Connective Tissue Growth Factor	1.13	0.5425
*DDR2*	Discoidin Domain Receptor Tyrosine Kinase 2	0.86	0.5965
*ENG*	Endoglin (CD105)	2.04	0.1706
*GATA 4*	GATA-Binding Factor 4	1.25	0.0533
*GJA1*	Connexin-43 (Gap Junction Protein Alpha 1)	0.88	0.2805
*GJA5*	Gap Junction Protein Alpha 5 (Connexin 40)	0.58	0.1210
*HCN1*	Hyperpolarization Activated Cyclic Nucleotide Gated Potassium Channel 1	0.40	0.0024
*HCN2*	Hyperpolarization Activated Cyclic Nucleotide Gated Potassium Channel 2	1.43	0.6689
*HCN3*	Hyperpolarization Activated Cyclic Nucleotide Gated Potassium Channel 3	0.95	0.9201
*HCN4*	Hyperpolarization Activated Cyclic Nucleotide Gated Potassium Channel 4	1.16	0.4744
*IRX4*	Iroquois Homeobox 4	0.96	0.7108
*JUP*	Junction Plakoglobin	1.01	0.9708
*KCNH2*	HERG1 - Ether-A-Go-Go-Related Gene Potassium Channel 1	1.12	0.6170
*KCNQ1*	Potassium Voltage-Gated Channel Subfamily Q Member 1 (Kv7.1)	1.87	0.1707
*MESP1*	Mesoderm Posterior BHLH Transcription Factor 1	2.34	0.3436
*MYC*	C-Myc	1.73	0.3395
*MYH6*	Myosin Heavy Chain 6	0.74	0.0216
*MYH7*	Myosin Heavy Chain 7	1.24	0.2239
*MYL2*	Cardiac Ventricular Myosin Light Chain 2 (MLC-2v)	1.53	0.1215
*MYL7*	Myosin Regulatory Light Chain 2, Atrial Isoform (MLC-2A)	0.69	0.0009
*NFATC1*	Nuclear Factor Of Activated T Cells 1	2.19	0.1411
*NKX2–5*	Cardiac-Specific Homeobox	1.19	0.1061
*NPPA*	Natriuretic Peptide A	1.78	0.0013
*NPPB*	Natriuretic Peptide B	1.64	0.0154
*PECAM1*	Platelet And Endothelial Cell Adhesion Molecule 1 (CD31)	1.55	0.1507
*PLN*	Phospholamban	1.37	0.0199
*POSTN*	Periostin	3.82	0.1770
*RYR2*	Ryanodine Receptor 2	0.82	0.0621
*SLC8A1*	Sodium/Calcium Exchanger 1 (NCX1)	1.09	0.1813
*TGFB1*	Transforming Growth Factor Beta 1	2.14	0.0262
*TNNI1*	Troponin I1, Slow Skeletal Type	0.98	0.9204
*TNNI3*	Troponin I3, Cardiac Type	1.90	0.0009
*TP53*	Tumor Protein P53	1.01	0.9708
*VIM*	Vimentin	0.86	0.2823
*WT1*	Wilms Tumor 1	0.56	0.1367

Listed alphabetically according to gene symbol. Expression level (fold change relative to control*) and *p* value for hCSC-supplemented hECTs normalized to hCM-only control hECTs

Considering all 59 genes investigated in the Nanostring custom panel, samples from the hCM-only control group clustered together in a principal component analysis, distinguishing them clearly from samples from the hCSC-supplemented group (Fig. 9g). Partial least squares regression analysis revealed several of the significantly upregulated genes that covary with twitch force parameters that were significantly higher in the hCSC-supplemented hECTs (Fig. 9h), suggesting that changes in these genes may have contributed to the increase in contractile function observed with hCSC supplementation. Specifically, the genes that clustered most closely with parameters associated with contractility (DF, +dF/dt, −dF/dt, and DS) were *PLN*, *NPPA*, and *TNNI3*, all three of which are centrally involved in the biological process of cardiac muscle contraction.

### Confirmatory functional studies using additional cell lines

**Fig. 10:**
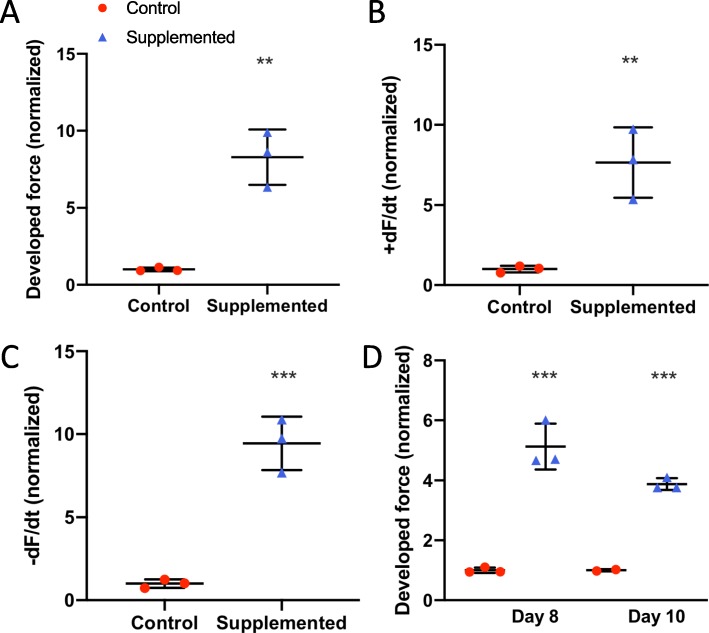
Contractile function using hCM from different hiPSC line. **a** Developed force, **b** rate of contraction (+dF/dt), and **c** rate of relaxation (−dF/dt), for hCM-only controls (red circle), and hCSC-supplemented hECTs (blue triangle), at day 6 after tissue fabrication. **d** Developed force at days 8 and 10 after tissue fabrication. Measurements performed at 1.25-Hz pacing frequency; values normalized to mean control hECT; *n* = 3 per group for all analysis except day 10, *n* = 2 for hCM-only controls, and *n* = 3 for hCSC-supplemented hECTs. Dot plots show individual data with bars representing mean ± SD; *p < 0.05, **p < 0.01, ***p < 0.001

We then performed functional studies using additional cell lines. We fabricated hECT to test the original hCSC line using hCM derived from a different hiPSC cell line (MSN02–4); as with our first functional experiments, we also observed a significant fold increase in DF for hCSC-supplemented vs control at day 6 (Fig. 10a), along with significantly higher +dF/dt (Fig. 10b) and −dF/dt (Fig. 10c). The significant effect on DF (Fig. 10d) persisted on days 8 and 10, as well as +dF/dt (*p* = 0.01 and *p* = 0.0004) and −dF/dt (*p* = 0.002 and *p* < 0.0001), during electrical pacing at 1.25 Hz. These hECTs displayed intrinsic (spontaneous) beat rate of 1.5 ± 0.02 Hz and 1.6 ± 0.47 Hz for control and hCSC-supplemented hECTs respectively at day 6; we therefore used a pacing frequency range beyond 1 Hz. The beat rate variability was also similar between hCSC-supplemented vs. hCM-only controls at matched time points (day 6: 0.09 ± 0.08 vs. 0.065 ± 0.02; day 8: 0.09 ± 0.05 vs. 0.05 ± 0.01; day 10: 0.07 ± 0.04 vs. 0.04 ± 0.003) (0.24 < *p* < 0.54).

**Fig. 11:**
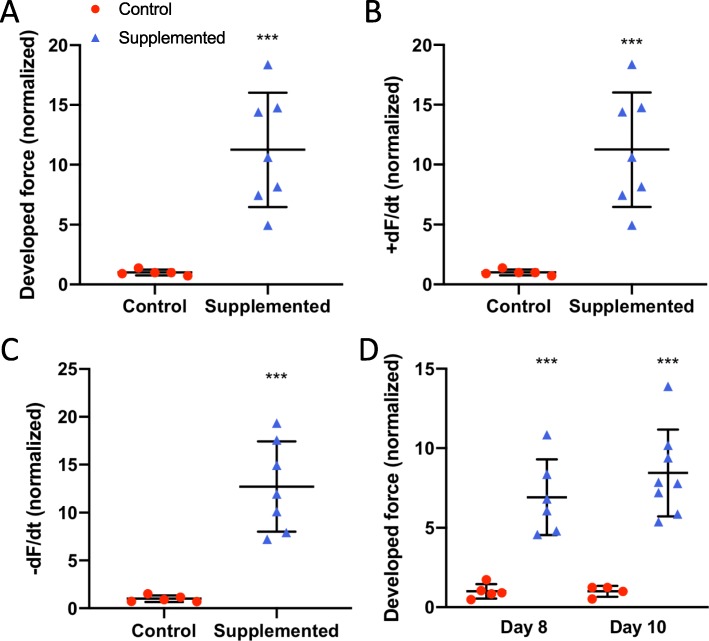
Contractile function using different hCSC line. **a** Developed force, **b** rate of contraction (+dF/dt), and **c** rate of relaxation (−dF/dt), for hCM-only controls (red circle) (*n* = 5), and hCSC-supplemented hECTs (blue triangle) (*n* = 7), at day 6 after tissue fabrication. **d** Developed force at day 8 (*n* = 5 and *n* = 6 respectively) and day 10 (*n* = 4 and *n* = 8 respectively) after tissue fabrication. Measurements performed at 1.0-Hz pacing frequency; values normalized to mean control hECTs. Dot plots show individual data with bars representing mean ± SD; ***p < 0.001

We then tested a different hCSC cell line. We fabricated hECTs using hCM derived from hiPSC cell line MSN02–4 and hCSCs from a second patient. And again, as observed with the first hCSC cell line, there was a significant fold increase in DF for hCSC-supplemented vs control at day 6 (Fig. 11a), along with significantly higher +dF/dt (Fig. 11b) and −dF/dt (Fig. 11c). The significant effect on DF (Fig. 11d) persisted on days 8 and 10, as well as +dF/dt (*p* = 0.0001 and *p* = 0.005) and −dF/dt (*p* < 0.0001 and *p* = 0.003) at 1.0 Hz pacing frequency. These hECT displayed an intrinsic (spontaneous) beat rate of 1.25 ± 0.06 Hz and 1.54 ± 0.1 Hz for control and hCSC-supplemented hECTs respectively at day 6; therefore, as in the case above, we used a pacing frequency range beyond 1 Hz. The beat rate variability was also similar between hCSC-supplemented vs. hCM-only controls at matched time points (day 6: 0.07 ± 0.03 vs. 0.09 ± 0.13; day 8: 0.15 ± 0.18 vs. 0.079 ± 0.09; day 10: 0.06 ± 0.03 vs. 0.05 ± 0.02) (0.41 < *p* < 0.62).

## Discussion

This study aimed to investigate the use of hCSCs to increase cardiac functional performance, using 3D human engineered cardiac tissue as a surrogate for human myocardium. Despite ongoing controversy regarding previously published data on the regenerative capacity of c-Kit^+^ cardiac stem cells [14, 15], recognized cardiotherapeutic advantages of hCSCs include T cell regulation to aid in mitigating the inflammatory response [39] and immunoprotection for potential use in allogeneic transplantation [40]. Combined with the positive results reported herein, including significant improvements in cardiac contractility without evidence of proarrhythmic risk, mounting data supports continued investigation of hCSC use for restoring cardiac function. In addition, our observed pro-maturation effects could be beneficial for in vitro applications of hiPSC-derived hCMs.

### hCSC supplementation improves hECT contractility without adversely affecting diastolic function or intrinsic electrical stability

We observed approximately twofold higher developed force in hCSC-supplemented hECTs compared to hCM-only controls, along with a roughly threefold increase in +dF/dt, −dF/dt, and developed stress. This is consistent with published preclinical studies that have reported significant CSC contributions to cardiac functional enhancement [5]. Such promising results, from both small and large animal models, have sometimes been contrasted with more modest outcomes in related clinical trials [6]. Indeed, the translational gap between preclinical and clinical studies remains a challenge. Here, for the first time, we confirmed the potential of hCSCs to augment cardiac function in a human-specific in vitro 3-D platform that may help to bridge this gap in clinical translation.

The donor characteristics, including age, stage of heart disease, and pharmacological therapies, can all affect the potential cardioprotective qualities of the CSCs [41]. This study used cells that were derived from the left ventricle apex biopsy of a 67-year-old male patient with advanced heart failure, obtained during left ventricular assist device (LVAD) implantation [33]. Based on recent evidence from the literature, one might have predicted minimal increase of contractility, since shortly after birth the cardioactive potential of CSCs and their secretome begins a steady decline with age [42]. Despite the donor’s age and condition, we observed 200 to 300% improvements in hECT contractility with just a 10% supplementation of hCSCs (vs. unsupplemented control hECTs). However, the functional benefits decreased in magnitude through the duration of the study, which is consistent with reports of short-term or transient benefits after administration of CSCs [7]. A variety of interventions have been suggested to enhance CSC potency and survival, including cell culture condition modifications [17], small molecules [43], drug interventions [41], and genetic modifications [44]. Such strategies might help extend the long-term benefits of hCSC treatment.

The passive force remained comparable for the two hECT groups through the time course of the study. In the myocardium, collagen deposition and fibrosis can often lead to a higher passive tension, which can ultimately lead to diastolic dysfunction [45]. The finding that active force increased significantly in hCSC-supplemented hECTs without elevating passive force supports the potential for hCSC treatment to improve systolic function without adversely affecting diastolic function.

**Fig. 12:**
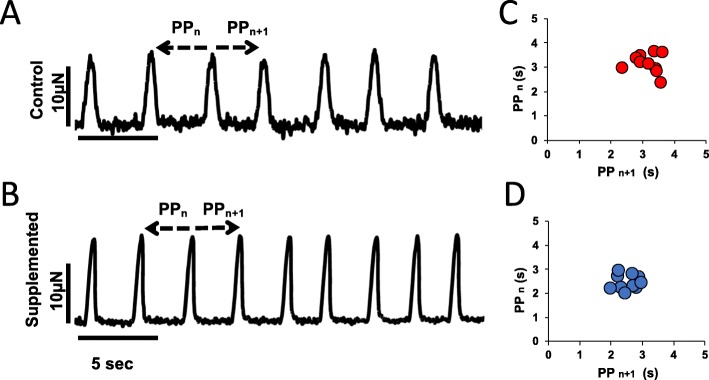
Twitch tracings under spontaneous beating. Representative twitch tracings of hCM-only control hECT (**a**) and hCSC-supplemented hECT (**b**); arrows point to measurements performed for each pair of consecutive twitches, PP_n_ = time to previous, and PP_n + 1_ = time to next. Corresponding Poincaré plots show the clustering of values according to the difference between each twitch in the time domain for hCM-only control hECTs (**c**) and hCSC-supplemented hECTs (**d**)

In spontaneously beating hECTs, while the overall rhythm is typically consistent for each tissue (Fig. 12a, b), there is intrinsic variability that can be quantified by the standard deviation of a sampling of twitches and calculation of the coefficient of variation (COV = SD/mean beat rate). The spontaneous beat rate was similar between the two hECT groups in this study; notably, we found no differences in spontaneous beat rate variability, with an average COV of about 15% for all conditions tested. The risk of arrhythmic events is of concern in stem cell studies [46], and the phenomenon of spontaneous beat rate variability is a potential indicator of a proarrhythmic substrate. For example, Schaaf et al. demonstrated increasing beat rate irregularity in engineered heart tissues exposed to escalating dosages of known proarrhythmic compounds [25]; recently, Mayourian et al. reported that supplementation of hECTs with human mesenchymal stem cells (hMSCs) can increase beat rate variability, which was related to arrhythmic potential using a computational model of heterocellular coupling between the hMSCs and hCMs [30]. A Poincaré plot serves as a graphical representation of this beat rate variability (Fig. 12c, d), where each twitch in a time series is represented by the time to the next beat on the *x*-axis, and time to the previous beat on the *y*-axis; higher dispersion of the plotted points indicates increased beat rate variability [25]. We observed no significant differences in beat rate variability between the control and hCSC-supplemented hECT groups in this study, which suggests minimal arrhythmic risk from hCSC supplementation. In addition, during pacing experiments, all hCSC-supplemented hECTs exhibited 1:1 capture up to 1.0 Hz pacing, fewer than half (8/17) of the hCM-only control hECTs were captured at the 1.0-Hz pacing frequency, suggesting improved electrophysiological coupling in tissues supplemented with hCSCs.

### hCSC supplementation improves hECT phenotypic and genotypic maturation

The microstructural composition of the hCSC-supplemented hECTs revealed better organization, alignment, and the appearance of better integration of adjacent cells, compared to control hECTs. This morphological difference is an indicator of improved maturation in the hCSC-supplemented hECTs [26, 47] and also can explain the improved functional outcomes, whereby the integration of the cells as a syncytium results in improved contractility. Furthermore, the functional studies showed that whereas hCM-only control hECTs exhibited a significant negative force-frequency relationship (FFR), the slope was nearly flat in hCSC-supplemented hECTs. This rectification indicates a progression toward a more mature and healthier cardiac phenotype that is typical of human myocardium. A negative FFR is common in failing myocardium, as well as in healthy but immature ventricular tissue, which transitions from a nearly flat FFR in newborns to a positive FFR in infants and older humans, which is characteristic of the healthy adult human heart [48]. In human engineered tissues in a biowire format, chronic treatment with electrical stimulation shifted the FFR from flat to positive, which was accompanied by increased expression levels of genes associated with ventricle maturation, including *NPPB* and *ACTA1* [23]. In our study, the upregulation of NPPB, ACTA1, and NPPA relative to control hECTs, along with other genetic indicators of maturation also accompanied the rectification of the FFR slope by hCSC supplementation. The hCSC-supplemented hECTs displayed higher ratios of *MHY7/MHY6*, *MYL2/MYL7*, and *TNNI3/TNNI1* relative to hCM-only controls, each of which is consistent with improved cardiomyocyte maturation, as discussed below.

In hCSC-supplemented hECTs, the elevated *MHY7/MH6* ratio resulted from a combination of an increase in *MHY7* expression and a decrease in *MYH6* relative to controls. The alpha and beta myosin heavy chains are essential to the sarcomeric contractile machinery in the heart, with *MHY6* more abundant in faster contracting and atrial myocardium, whereas *MHY7* is associated with slower contracting and more energy-efficient muscle and is the predominant isoform expressed in the healthy left ventricle (LV) [49]. In humans, the ratio of *MHY7/MH6* increases with age in the LV [50], as the levels of *MHY6* decrease [51]. hCMs with extended time in culture [21], and engineered cardiac tissues cultured in conditions that maximized structural and functional maturation [26] have also shown this trend.

The myosin light chain isoform ratio *MYL2/MYL7* was also significantly higher in hCSC-supplemented hECTs relative to controls. *MYL2* (also known as *MLC2v*) is a marker of mature ventricular cardiomyocytes, while *MYL7* (or *MLC2a*) serves as a marker of atrial myocytes but also of immature ventricular myocytes [18]. Thus, an increase in *MYL2/MYL7* (i.e., *MLC2v/MLC2a*) is a feature of hCM maturation [22] and suggests elevated maturation in hCSC-supplemented hECTs.

The *TNNI3/TNNI1* ratio also tended to be higher in hECTs supplemented with hCSCs. *TNNI1* is the troponin subtype mainly expressed in immature cardiomyocytes, whereas *TNNI3* is a marker for adult cardiomyocytes [19, 21]. Others have reported that hCM gene expression levels of *TNNI3* and phospholamban (*PLN*) increase with time in culture [18], and maturation of hCM-engineered heart tissues was associated with upregulation of *TNNI3*, *MYL2*, and *PLN* [47]; in our hCSC-supplemented hECTs, *TNNI3* and *PLN* were also significantly upregulated relative to controls.

DEGs elevated in hCSC-supplemented versus control hECTs also included *TGFB1*, *COL1A1*, and *COL3A1*, genes associated with extracellular matrix regulation that can have both beneficial and cautionary implications. For example, the role of TGFB in the heart is pleiotropic [52]. Inhibition of *TGFB1* can produce disparate effects on mortality rate and infarct size after MI in mice, depending on the timing of inhibition [53]. Finally, observed upregulation of *COL1A1* and *COL3A1* in hCSC-supplemented hECTs suggests hCSCs could impact remodeling of the cardiac extracellular matrix (ECM). Whereas excessive ECM deposition can be functionally detrimental [52], the hCSC-treated hECTs exhibited superior functional outcomes compared to hCM-only control hECTs.

The indicators of maturation from the genetic analysis are not proposed to indicate trans-differentiation of hCSCs into cardiomyocytes. Rather, we suggest that the existing hiPSC-derived CMs developed toward a more mature genotype in the presence of cardiac stem cells, which was accompanied by the observed improvements in cardiac function, as might be reasonably expected.

### Limitations

In our hECT model, the hCSCs interacted with cardiomyocytes and were functionally tested in a human species-specific, 3D cardiomimetic environment, which provides a more translatable in vitro setting than the traditional 2-D culture format. Despite the significant improvement in maturation provided by hCSC supplementation, the resulting hECTs and their constituent hiPSC-derived CMs did not transform into adult-like myocardium. Studies report important advances in the active research area of achieving a more advanced maturation and native cardiac structure and function [26, 39, 47, 54]. While this may be critical for surgical implantation applications, we and others have shown that despite sub-physiologic levels of force generation, hECTs are capable of reproducing a wide range of healthy [20, 23, 24] and diseased cardiac conditions [26–28] as noted earlier. Therefore, it is reasonable that the outcomes we reported could help decipher the complex role that hCSCs play in the native cardiac setting. More studies will be necessary to explain the mechanisms through which the hCSCs influenced the phenotypic and genotypic maturation.

While our hECT model is suitable for in vitro investigation of contractility, with advantages of providing a 3-D human species-specific model, the native myocardium is a more complex tissue, which 3D bioprinting technology aims to capture [55, 56]. Nevertheless, unlike healthy heart muscle, failing myocardium reactivates aspects of the fetal gene program that have similarities to immature hiPSC-derived cardiomyocytes, and therefore the findings from our hECT model may still have relevance in the context of heart failure. In addition, preclinical and clinical cell therapy approaches involve the delivery of therapeutic cells into the in vivo host myocardium, which differs from our hECT fabrication process. Nevertheless, the hECT model was able to recapitulate several key observations from in vivo animal studies, and our findings support further investigation of the cardiotherapeutic potential of hCSCs. In this study, the cell fate of hCSCs was not investigated in detail. While it has been suggested that cardiac stem cells have endothelial-like or vasculogenic effects [14, 15], we could not confirm neovessel formation within the hECTs. In the future, hECT fabricated with trilineage cardiac cells derived from hiPSCs [54, 57] could be useful for such an investigation. Another limitation was the short evaluation period, which was also a constraint in our recent work using hECT supplemented with mesenchymal stem cells [30]. Investigations into longer-term phenomena related to cell fate and survival, and potential cardiac remodeling in response to hCSC treatment are still needed.

## Conclusions

In conclusion, our study is the first to show that hCSC treatment is beneficial in an in vitro hECT model of human myocardium. Replacing 10% of the cell composition with hCSCs conferred a significant increase in contractile function, without adverse effects on diastolic force or electrical stability. A more mature cardiac genotype was also observed, accompanied by rectification of the force-frequency slope, within 6 days of hCSC treatment.

While the reparative potential of hCSCs has been reported to decrease with donor age, this study found a significant improvement in contractile performance using hCSCs that originated from a patient with advanced age and severe cardiac pathology. This suggests an intriguing possibility that myocardial tissue harvested at the time of LVAD intervention in patients with advanced heart failure could serve as a source of cardiac stem cells with cardioactive characteristics for subsequent autologous stem cell-mediated cardiotherapies.

## Supplementary information

**Additional file 1: Figure S1.** Representative image of hCSC-supplemented hECT fabricated with hCSCs labeled with Qtracker-655 and co-stained with cardiac troponin T. A) Confocal max projection image showing cardiac marker Troponin T (green), hCSCs labeled with Qtracker-655 (magenta), and nuclei stained with DAPI (blue); arrowheads point to the presence of Qtracker, scale bar = 50 μm. B, C) Zoom in of inset from panel A. B) Is a Max projection of with orthogonal views, scale bar = 5 μm. C) Single slice of the same inset, with orthogonal views. The orthogonal views of the max projection (in B) and the single slice (in C) show that there is no colocalization of Qtracker and Troponin T; where Qtracker positive cells appear adjacent but distinct from troponin T positive cells.

## Data Availability

The datasets generated during and/or analyzed during the current study are available from the corresponding author on reasonable request.

## Abbreviations

hCSCs: Human cardiac stem cells
hECTs: Human engineered cardiac tissues
hiPSCs: Human induced pluripotent stem cells
hCMs: Human cardiomyoctes
CSA: Cross-sectional area
DF: Developed force
DS: Developed stress
+dF/dt: Maximum rate of contraction
−dF/dt: Maximum rate of relaxation
COV: Coefficient of variation
PP: Peak to peak
PDMS: Polydimethylsiloxane
PBS: Phosphate-buffered saline
PLSR: Partial least squares regression
PCA: Principal component analysis
