# Supplementary figures and images for: Adult human cardiac stem cell supplementation effectively increases contractile function and maturation in human engineered cardiac tissues
Source: Stem Cell Res Ther. 2019 Dec 4;10:373. doi: 10.1186/s13287-019-1486-4 (PMC6894319; doi:10.1186/s13287-019-1486-4)

A

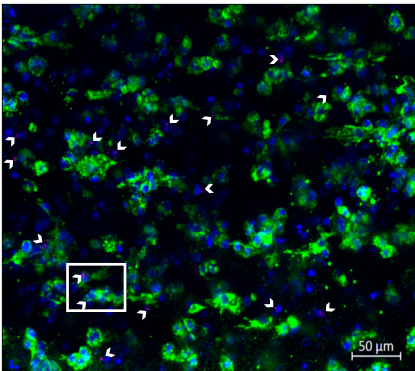

B

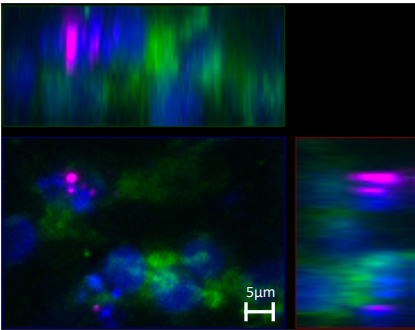

C

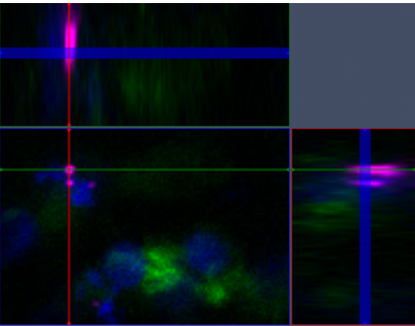

Supplement: Supplementary file 1 — Additional file 1: Figure S1. Representative image of hCSC-supplemented hECT fabricated with hCSCs labeled with Qtracker-655 and co-stained with cardiac troponin T. A) Confocal max projection image showing cardiac marker Troponin T (green), hCSCs labeled with Qtracker-655 (magenta), and nuclei stained with DAPI (blue); arrowheads point to the presence of Qtracker, scale bar = 50 μm. B, C) Zoom in of inset from panel A. B) Is a Max projection of with orthogonal views, scale bar = 5 μm. C) Single slice of the same inset, with orthogonal views. The orthogonal views of the max projection (in B) and the single slice (in C) show that there is no colocalization of Qtracker and Troponin T; where Qtracker positive cells appear adjacent but distinct from troponin T positive cells. [file 13287_2019_1486_MOESM1_ESM.pdf]
